# Supplementary material for: Impact of periprocedural morphine use on mortality in STEMI patients treated with primary PCI
Source: PLoS One. 2021 Jan 13;16(1):e0245433. doi: 10.1371/journal.pone.0245433 (PMC7806148; doi:10.1371/journal.pone.0245433)
Supplement: S1 Appendix — (PDF) [file pone.0245433.s001.pdf]

S1 Appendix

Extended Methods: Statistical analysis.

For descriptive statistics, variables in 2×2 contingency tables were assessed using Fisher's exact test. Categorical data in 2×k tables were analyzed using the unordered chi-squared test or, to detect linear trend, the chi-squared test for trend (Cochran-Armitage test). Continuous parameters were examined for normality with the D'Agostino-Pearson and Shapiro-Wilk tests. As none of the continuous variables showed normal distribution, the Wilcoxon rank sum test was applied for comparisons. A two-tailed p value less than 0.05 was considered statistically significant.

To adjust for confounders, two distinct propensity score-based techniques were applied [1]. We used 1 to 1 nearest neighbor propensity score matching with a caliper width of 0.2 of the standard deviation of the logit of the propensity score to estimate the average treatment effect for the treated (ATT) yielding a total of 728 cases [2]. In addition, we also assessed the average treatment effect (ATE) by inverse probability of treatment weighting (IPTW) using stabilized weights retaining data from all patients [1,3]. The propensity score model was constructed using logistic regression including all measured baseline covariates listed in Table 1 that could affect treatment assignment and / or are known to be associated with the primary end point (all-cause mortality). Presence of non-linear relationships of the continuous variables to log odds of treatment with morphine were explored using restricted cubic splines (RCS) which were evaluated graphically and by formal Wald testing for linearity. Since onset-to-door time (time from onset of symptoms to arrival at the PCI center) has been proved to be non-linearly associated with the logit of treatment assignment, it was represented by a RCS with five knots technically resulting in four variables. Balance on baseline covariates between the treated and control groups was evaluated using absolute standardized differences [4]. A value less than 0.1 was considered as an acceptable standardized bias.

Absolute risk differences in all-cause mortality were captured by Kaplan-Meier survival curves which were compared using the log-rank test, log-rank test stratified on matched pairs, and the design-based log-rank test for the original, matched, and weighted samples, respectively. The relative change in the hazard of death was estimated using univariable Cox models as suggested by Austin [1,5]. To account for the matched nature of the PSM sample, a robust variance estimator was used, whereas the naïve model-based estimate was applied for the original sample [6]. For the stabilized ATE-weighted model we calculated the bootstrap estimate of the standard error using 10000 resamples as described by Austin [3]. Assumption of proportional hazards was evaluated graphically and by formal testing. Ties were handled by Efron's approximation. Statistical significance was calculated using the Wald test, as it does not assume independence of observations within a cluster.

As to the secondary outcome measure, distributions of predischarge LVEFs in the treated and control groups were compared by the Wilcoxon rank sum test (Mann-Whitney test), the Wilcoxon signed rank test, and the design-based Wilcoxon test in the original, matched, and weighted samples, respectively.

All statistical analyses and graphical interpretation of the results were carried out with R version 4.0.2 (R Foundation for Statistical Computing, Vienna, Austria) using the boot 1.3-25, cobalt 4.2.2, coin 1.3-1, fBasics 3042.89.1, ggplot2 3.3.2, MatchIt 3.0.2, survey 4.0, rms 6.0-1, survival 3.2-3, survminer 0.4.8, and WeightIt 0.10.1 packages.

References

1. Austin PC. The performance of different propensity score methods for estimating marginal hazard ratios. Stat Med. 2013;32:2837–2849. doi:10.1002/sim.5705

2. Austin PC. Optimal caliper widths for propensity-score matching when estimating differences in means and differences in proportions in observational studies. Pharm Stat. 2011;10:150–161. doi:10.1002/pst.433

3. Austin PC. Variance estimation when using inverse probability of treatment weighting (IPTW) with survival analysis. Stat Med. 2016;35:5642–5655. doi:10.1002/sim.7084

4. Austin PC. Balance diagnostics for comparing the distribution of baseline covariates between treatment groups in propensity-score matched samples. Stat Med. 2009;28:3083–3107. doi:10.1002/sim.3697

5. Austin PC. The use of propensity score methods with survival or time-to-event outcomes: reporting measures of effect similar to those used in randomized experiments. Stat Med. 2014;33:1242–1258. doi:10.1002/sim.5984

6. Lin DY, Wei LJ. The Robust Inference for the Cox Proportional Hazards Model. J Am Stat Assoc. 1989;84:1074–1078. doi:10.1080/01621459.1989.10478874
